# SARS-CoV-2 Neutralizing Antibodies in Free-Ranging Fallow Deer (*Dama dama*) and Red Deer (*Cervus elaphus*) in Suburban and Rural Areas in Spain

**DOI:** 10.1155/2023/3324790

**Published:** 2023-08-17

**Authors:** Paloma Encinas, Alba Escalera, Teresa Aydillo, Irene Iglesias, Martha I. Nelson, Adolfo García-Sastre, Gustavo del Real

**Affiliations:** ^1^Department of Biotechnology, National Institute of Agricultural and Food Research and Technology (INIA/CSIC), Madrid, Spain; ^2^Department of Microbiology, Icahn School of Medicine at Mount Sinai, New York, NY, USA; ^3^Global Health and Emerging Pathogens Institute, Icahn School of Medicine at Mount Sinai, New York, NY, USA; ^4^Graduate School of Biomedical Sciences, Icahn School of Medicine at Mount Sinai, New York, NY, USA; ^5^Center for Animal Health Research, INIA-CISA/CSIC, Valdeolmos, Madrid, Spain; ^6^National Center for Biotechnology Information, National Library of Medicine, National Institutes of Health, Bethesda, MD, USA; ^7^Department of Medicine, Division of Infectious Diseases, Icahn School of Medicine at Mount Sinai, New York, NY, USA; ^8^The Tisch Cancer Institute, Icahn School of Medicine at Mount Sinai, New York, NY, USA; ^9^Department of Pathology, Molecular and Cell-Based Medicine, Icahn School of Medicine at Mount Sinai, New York, NY, USA

## Abstract

Effective control of zoonotic infectious diseases requires identifying the animal species involved in the infectious cycle as transmitters or reservoirs where the pathogen could persist and evolve, increasing the risk of reintroduction of new variants in humans and animals. Multiple surveillance studies have detected the presence of severe acute respiratory syndrome coronavirus-2 (SARS-CoV-2) or specific antibodies in free-ranging white-tailed deer (*Odocoileus virginianus*) in North America, being the wild mammal species with the most reported cases of SARS-CoV-2 infection. However, so far, all attempts to detect the infection in European cervids have been unsuccessful. In this study, we demonstrated the presence of specific antibodies against SARS-CoV-2 in European fallow deer (*Dama dama*) and red deer (*Cervus elaphus*) in Spain. However, all samples of European roe deer (*Capreolus capreolus*), taxonomically related to the American white-tailed deer, were seronegative for the virus. We tested 215 serum samples from roe deer (*n* = 116), red deer (*n* = 63), and fallow deer (*n* = 36) collected in eight provinces of central-southern Spain between 2017 and 2022. We first screened sera by a surrogate virus neutralization test (sVNT) ELISA based on the binding of the receptor-binding domain of SARS-CoV-2 to the angiotensin-converting enzyme 2 (ACE2) receptor and then confirmed positive samples by a conventional virus neutralization test (cVNT) against the Alpha, Delta, and Omicron variants. Four fallow deer and two red deer samples were seropositive in both neutralization assays. Four samples of fallow deer and one of red deer, collected in a suburban park near Madrid in February 2022, had antibodies against the Alpha, Delta, and Omicron variants, while a seropositive sample of red deer, collected in a rural area in February 2021, was specific for the Delta variant. All samples collected before the start of the pandemic in Spain were seronegative for sVNT, which also indicates that there are not cross-reactive antibodies potentially elicited by other viruses antigenically related to SARS-CoV-2. The results indicate probable human-to-deer transmission of SARS-CoV-2, but do not clarify whether there was subsequent spread within herds.

## 1. Introduction

Severe acute respiratory syndrome coronavirus-2 (SARS-CoV-2), the etiologic agent of coronavirus disease 2019 (COVID-19), was first reported in humans in December 2019 in China and subsequently became pandemic. SARS-CoV-2 belongs to the genera Betacoronavirus (subgenus Sarbecovirus) that, together with the Alpha-, Gamma-, and Deltacoronaviruses, comprise the family Coronaviridae [1]. They are positive-sense single-stranded RNA viruses and have a lipid envelope [2]. Other virulent human Betacoronaviruses are SARS-CoV, emerged in 2002, and Middle East respiratory syndrome coronavirus (MERS-CoV) in 2012 and are considered to be a zoonosis. SARS-CoV is not circulating in humans anymore, while small number of human MERS-CoV cases continue to be reported as a result of transmission from infected camel to humans, and SARS-CoV-2 has become endemic in humans. SARS-CoV-2 also had a zoonotic origin, most probably from bats [3], although it became pandemic due to human-to-human transmission. Bats are also the main natural reservoir of SARS-CoV and MERS-CoV and most likely for SARS-CoV-2 [3]. In addition, intermediate animal hosts play an essential role in transmission to humans, civet cats for SARS-CoV, and in the case of MERS-CoV, camels constitute a natural reservoir where the viruses remain and maintain the zoonotic risk. Regarding SARS-CoV-2, reverse zoonotic transmission events involving mainly companion, farm, and zoo animals have been reported [4–9]. A public database has recently been created that collects reports of infected mammals worldwide [10]. This database is growing steadily and will presumably help to better understand the epidemiology of the virus infection and its evolution in the human/animal interface in different ecosystems. According to the last update of that database (February 2023), 744 SARS-CoV-2 animal events have been reported in 31 animal species of 39 countries.

Surveillance of SARS-CoV-2 in wild and domestic animals is of utmost importance to assess their role in COVID-19 epidemiology, as well as animal health implications. The spillover of SARS-CoV-2 infection from humans to free-living or domestic animals could create new reservoirs in which the virus might persist and evolve independently, as well as give rise to new variants with zoonotic potential. For example, reciprocal transmission has been documented between farm minks and farmers or between hamsters and a pet shop worker [8, 11]. Therefore, it is crucial to know if the virus spreads among other species of mammals and whether they could transmit it to humans. Some of the susceptible species become infected due to frequent or close contact with humans, such as pets or farm animals, but others, such as suburban wildlife (deer and mice); the route of infection remains to be elucidated. Comparative molecular analysis of angiotensin-converting enzyme 2 (ACE2) receptor, used by SARS-CoV-2 to enter cells [12], showed that ACE2 receptor is highly conserved among mammalian species, including humans, indicating the potential susceptibility of multiple species to infection with SARS-CoV-2 [13].

Multiple surveillance studies have detected the presence of SARS-CoV-2 or specific antibodies in free-ranging white-tailed deer (WTD, *Odocoileus virginianus*) in the U.S. and Canada, being the wild mammal species with the most published cases of SARS-CoV-2 infection [14–19]. This free-ranging ruminant species is the predominant cervid in the U.S. where it is abundant in rural, suburban, and urban areas, including areas densely populated by humans, where easy interactions could facilitate reciprocal pathogen transmissions [16, 20–23]. Infection spread in deer populations is quite feasible given the social behavior of these species and their high mobility. It was found that 33.2% of samples from free-living and captive deer in Iowa were positive for SARS-CoV-2 by RT-PCR and the rate of positive sample raised to 82.5% following the peak of human cases in November 2020 and coincided with the deer hunting season [16]. Although there are no studies on virus transmission among free-ranging deer, deer-to-deer transmission by direct contact has been shown in experimentally infected adult and juvenile animals [21]. All this data strongly suggested multiple human-to-deer transmissions followed by subsequent spread within the herds. Early surveillance studies have shown that SARS-CoV-2 variants found in deer mirror those circulating in nearby human populations. Whole genome sequencing of the isolated viruses identified 12 SARS-CoV-2 lineages homologous to those circulating in humans [16]. The amino acid sequences of WTD and human ACE2 are quite similar and the receptor of both efficiently bind to the SARS-CoV-2 spike protein [24], which would explain this high efficiency of inter- and intraspecific transmission. Worth mentioning that the ACE2 of all three deer species in the U.S., WTD, reindeer (*Rangifer tarandus*), and Pere David's deer (*Elaphurus davidianus*) were predicted to have high binding with SARS-CoV-2 spike [12].

Field studies suggest sustained horizontal transmission among deer in the wild, posing the risk of deer becoming a reservoir for SARS-CoV-2 and a source of infections for other species, including humans [25, 26]. As an example, camels became reservoir for the MERS-CoV and occasionally transmit the virus to people. So far, however, too few cases of deer-to-human transmission have been reported to consider deer as reservoir for human infection [27]. Nevertheless, it remains unknown what is the long-term impact on the epidemiology of infection in humans and other animal species. Therefore, further research is needed to explore other scenarios in wildlife or livestock where deer's viruses might spread and evolve.

Given the high prevalence of SARS-CoV-2 infection in North American deer, numerous attempts to search for the virus in European deer have been made, but have so far been unsuccessful. SARS-CoV-2 surveys conducted in Germany and Austria during 2020–2022 resulted negative in European roe deer (ERD, *Capreolus capreolus*), red deer (RD, *Cervus elaphus*), and fallow deer (FD, *Dama dama*) [28, 29]. Similarly, serological surveys in UK, conducted from January 2020 to May 2021, resulted negative [30], as well as in Belgium [31].

Comparative studies of ACE2 receptor do not reveal significant differences between several deer species, suggesting that additional deer species may be likewise susceptible to SARS-CoV-2 infections [11]. Therefore, *a priori*, there should be no reason why European deer do not become infected with SARS-CoV-2.

**Figure 1:**
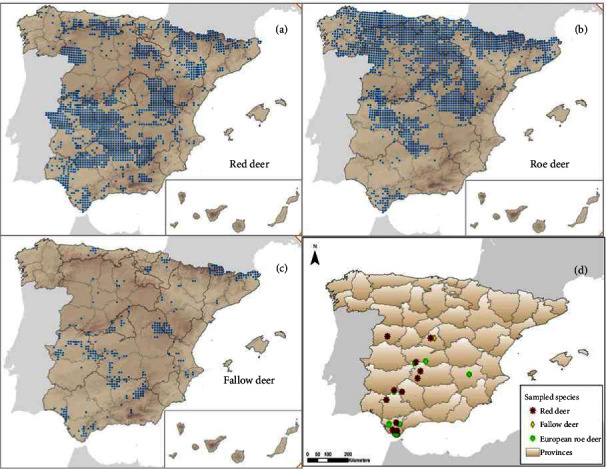
Distribution (a–c) and sampling locations (d) of red deer, roe deer, and fallow deer in Spain.

To investigate whether the same could happen in cervid populations in Spain, we carried out a serological survey of cervid populations in different rural and suburban regions in Spain (Figure 1). As in the rest of Europe, the most common cervid species in Iberian Peninsula are the RD, ERD, and the FD [31]. The ERD and the North American WTD are taxonomically related as members of the subfamily Capreolinae of the Cervidae family [32]. These free-ranging cervids are mainly distributed in forested areas of Spain, although populations are also found in suburban areas. In addition, in recent years, there has been great interest in the pseudodomestication and captive breeding of these species for hunting purposes [33, 34].

Serological methods are, in many cases, the most adequate and reliable diagnostic tools for the surveillance of infectious diseases, especially in free-ranging animals. Specific antibodies persist for long periods and there is a wide variety of techniques with high sensitivity, specificity, and low cost [35]. In addition, deer have been shown to elicit a rapid and robust neutralizing antibody response upon experimental infection [36].

## 2. Materials and Methods

A total of 215 serum samples from ERD (*n* = 116), RD (*n* = 63), and FD (*n* = 36) were collected at random in eight provinces of central-southern Spain (Figure 1(d)) in two periods, prepandemic (*n* = 69): 2017 (*n* = 9 ERD), 2018 (*n* = 12 ERD), and 2019 (*n* = 24 ERD, 19 RD, 5 FD) and pandemic (*n* = 146): 2020 (*n* = 18 ERD, 14 RD, 18 FD), 2021 (*n* = 23 ERD, 15 RD, 3 FD), and 2022 (*n* = 30 ERD, 15 RD, 10 FD) (Table 1). Samples from FD were only collected in Madrid province. The prepandemic samples used in this study were from frozen serum collections taken for routine wildlife health surveys. The serum samples used in this study were collected from legally hunted and captured animals in accordance with Spanish and European regulations. Ethical approval from an Institutional Animal Care and Use Committee was not considered necessary. Blood samples were collected from hunted animals using a standardized method [37]. After blood extraction, sera from clotted blood were collected and frozen until use. Before serological assays were performed, sera were heated to 55°C for 30 min to avoid unspecific reactions. At the time of sampling, animals showed no appreciable clinical signs. There are not clinical studies on SARS-CoV-2 infection in free-ranging deer but experimentally infected captive deer develop mild clinical symptoms and a high proportion of infected deer remain asymptomatic [22].

Sera were assayed by a SARS-CoV-2 Surrogate Virus Neutralization Test (sVNT) Kit (GenScript, NJ, USA) consisting in a competition ELISA based on the binding of the receptor-binding domain (RBD) of SARS-CoV-2 to its human host cell receptor, ACE2 [38]. sVNT was used according to the manufacturer's instructions, but a corrected inhibition value of 30% was adopted as the cutoff instead of the 20% recommended by the kit, consistent with values obtained with samples from confirmed seronegative deer. To confirm the sVNT-positive samples, they were then tested by conventional virus neutralization tests (cVNT). The United States Department of Agriculture definition of a confirmed SARS-CoV-2 case in animals requires a positive cVNT result. Therefore, cVNT were performed against three authentic SARS-CoV-2 variants (Alpha, Delta, and Omicron BA.1 subvariant). Experiments were performed as previously described following the appropriate guidelines in the Biosafety Level 3 (BSL-3) containment facility at the Department of Microbiology of Icahn School of Medicine at Mount Sinai (ISMMS) in New York (USA). To *in vitro* assess the levels of neutralizing antibodies against these variants, a SARS-CoV-2 infection permissive VeroE6 cell line stably expressing TMPRSS2 protease was used. Importantly, this transmembrane protease has been shown to be required for efficient SARS-CoV-2 spike protein processing and subsequent viral infection [39–41]. Briefly, VeroE6-TMPRSS2 cells (BPS Biosciences) were seeded at a density of 30,000 cells per well in a 96-well plate and cultured according to manufacturer's instructions. The following day, heat-inactivated serum samples (dilution of 1 : 10) were serially diluted threefold in 1x minimum essential medium (MEM) with 2% fetal bovine serum (FBS). The authentic SARS-CoV-2 variants were diluted to a concentration of 600 Tissue Culture Infectious Dose 50% (TCID50) in 1x MEM. Then, 80 *µ*l of serum dilution and 80 *µ*l of virus dilution were added to a new 96-well plate and incubated for 1 hr at 37°C. Next, media was removed from Vero-TMPRSS2 cells and 120 *µ*l of virus–serum mixture was added to the cells. The cells were incubated at 37 °C for 1 hr. After 1 hr incubation, virus–serum mixture was removed from the cells and 100 *µ*l of the corresponding serum dilutions and 100 *µ*l of 1x MEM with 2% FBS were added to the cells. Then, cells were incubated for 24 hr for the Alpha and Delta variants or 48 hr for the Omicron BA.1 variant. After incubation, cells were fixed with 10% paraformaldehyde (Polysciences) for 24 hr at 4°C. Following fixation, cells were washed with phosphate-buffered saline (Corning) with Tween-20 (Fisher) (PBST) and permeabilized with 0.1% Triton X-100 (Fisher) for 15 min at room temperature (RT). The cells were washed three times with PBST and blocked with 3% milk in PBST for 1 hr at RT. Then, cells were incubated with anti-SARS nucleocapsid antibody (1C7C7, kindly provided by Dr. Moran at ISMMS) at a dilution of 1 : 1,000 in 1% milk in PBST and incubated for 1 hr at RT. The cells were washed three times with PBST. Then, cells were incubated with goat antimouse immunoglobulin G-horseradish peroxidase (IgG-HRP) (Abcam, Cat. ab6823) at a dilution of 1 : 10,000 in 1% milk in PBST and incubated for 1 hr at RT. The cells were washed three times with PBST and TMB ELISA peroxidase substrate (Rockland) was added. After 15 min incubation, sulfuric acid 4.0 N (Fisher) was added to stop the reaction and the readout was done using a Synergy H1 plate reader (BioTek) at an OD 450. Neutralizing antibodies titers were plotted as reciprocal dilution of serum and percentage of inhibition of virus using GraphPad Prism 9. Nonlinear regression curve fit analysis over the dilution curve was performed to calculate the half-maximum inhibitory concentration (IC50) values.

## 3. Results and Discussion

**Figure 2:**
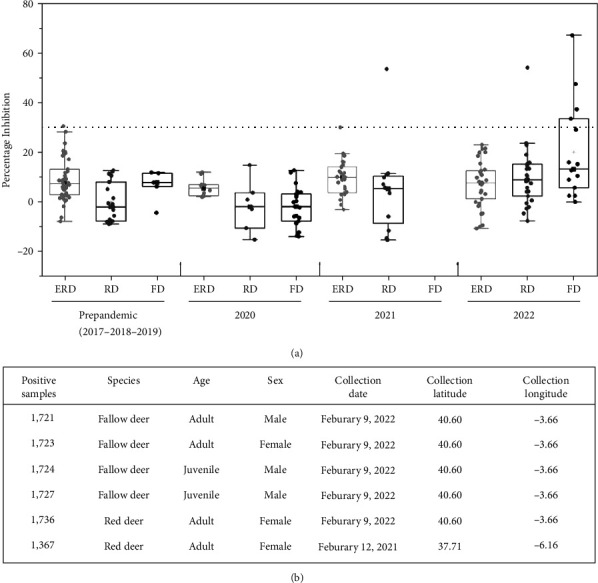
(a) SARS-CoV-2 neutralization (sVNT) by sera from European roe deer (ERD), red deer (RD), and fallow deer (FD) collected in Spain before and during the pandemic. Serum titers expressed as percentage inhibition of ACE2 binding to virus receptor (RBD). Dashed line is the cutoff value (30%). Boxes represent the interquartile range 25–75, horizontal bars are medians, asterisks are means, and vertical bars are the maximum and minimum values; outliers. (b) Positive sample data. sVNT, surrogate virus neutralization test; ACE2, angiotensin-converting enzyme 2; RBD, receptor-binding domain.

**Figure 3:**
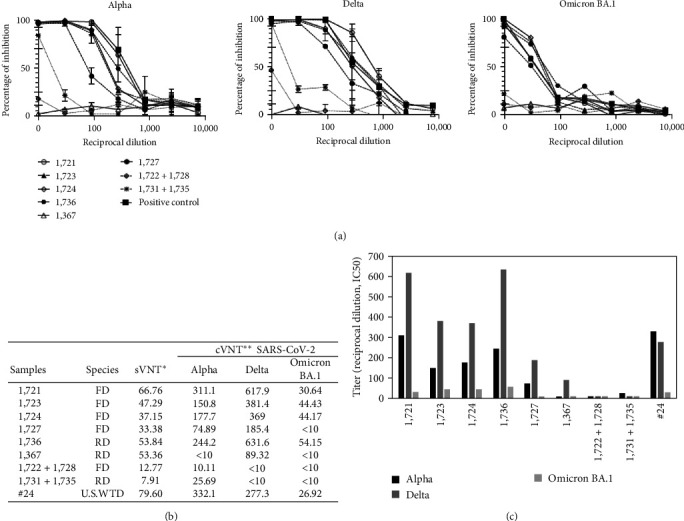
(a) Neutralization (cVNT) profile of SARS-CoV-2 Alpha, Delta, and Omicron BA.1 variants by antisera from fallow deer (FD) and red deer (RD) in Spain. Experiments were perfomed in duplicates for Alpha and Delta and as single replicate for Omicron variant due to limited amount of sample. Shown are reciprocal dilution for each sample and percentage of inhibition of virus. (b) Serum titers expressed as IC50 values (cVNT) and as inhibition percentage (sVNT) of seropositive FD (1,721, 1,723, 1,724, 1,727) and RD (1,736, 1,367), positive control American white-tailed deer (U.S. WTD #24), and negative controls (FD 1,722 + 1,728, RD 1,731 + 1,735). Samples under limit of detection were assigned a value of <10. (c) Bar chart of IC50 titers for each serum sample and SARS-CoV-2 variant.

All samples collected during the prepandemic period (2017–2019) were negative for sVNT. Six seropositive samples (% inhibition >30%) collected during the pandemic period, two from RD (53.8%, 53.3%) and four from FD (66.7%, 47.3%, 37.1%, 35.4%), were identified (Figure 2(a)). Positive and negative sera from American WTD were used as controls. Five of the six positive samples were collected on February 9, 2022 and one on February 12, 2021. Three of the six seropositive samples were from females and three from males, four were adult, and two juvenile animals (Figure 2(b)), but further research with more samples should be done to assess the influence of animal sex and age on infection. Due to the limited amount of serum available from some samples, the six positive sera were confirmed by cVNT only against the predominant variants of SARS- CoV-2 in the successive waves of the pandemic during 2021 and 2022 in Spain (Alpha, Delta, and Omicron) (Figure 3). Neutralization profile of each sample is shown in Figure 3(a), and the titer values (IC50) are shown in Figures 3(b) and 3(c). All samples in this study seropositive for sVNT were also positive for cVNT, in contrast to other studies that reported deer sera with cross-reactive antibodies against SARS-CoV-2 RBD, with a positive result for sVNT but negative for cVNT, probably induced by another deer coronavirus antigenically related to SARS-CoV-2 [28].

The prevalence found in our study was 4.1% (6/146; 95% CI 0.9–7.3) of all cervid samples collected during the pandemic years (2020–2022); five of the six positive samples were collected in 2022 and one in 2021. Therefore, the annual prevalence was 0.0% (0/50) for 2020, 2.4% (1/41) for 2021, and 9.1% (5/55) for 2022. Notably, a prevalence of 11.9% (5/42; 95% CI 2.1–21.7) vs. 0.9% (1/104; 95% CI 0.0–2.8) of samples collected in suburban and rural areas, respectively. The prevalence, according to deer species, was 4.5% (2/44; 95% CI 1.6–10.7) in RD and 12.9% (4/31; 95% CI 1.1–24.7) in FD. As mentioned before, serological studies with a larger number of samples should be performed to confirm these data.

**Figure 4:**
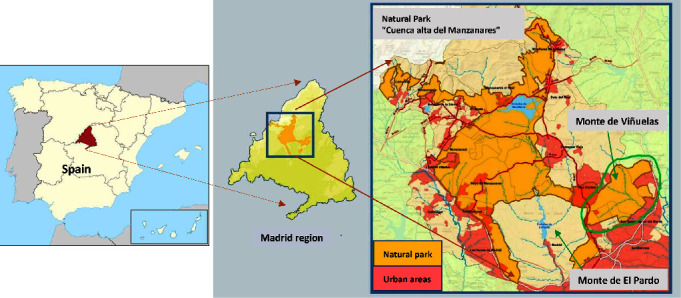
Map of Monte de Viñuelas (circled in green) where seropositive fallow deer and red deer samples were collected.

It should be noted that five of the six positive serum samples were collected in Monte de Viñuelas (40°36′31″N, 3°38′47″W), a 3,000 ha fenced natural park adjacent to Monte de El Pardo, and another 15,100 ha fenced natural park located to the north of the city of Madrid and which is part of the “Cuenca Alta del Manzanares” regional park, in the province of Madrid (Figure 4). These parks are directly surrounded by important human populations with more than 3.55 million inhabitants and a density of 3,500 inhabitants/ha. Both natural parks have abundant wildlife, especially ungulates such as RD, FD, and wild boar. Cervid species that share habitat in this park adopt gregarious habits and have higher densities than in rural areas (1.0–1.7/ha). Generally, they behave as animals as in other areas where hunting is not allowed, facilitating the transmission of pathogens within the herd.

**Figure 5:**
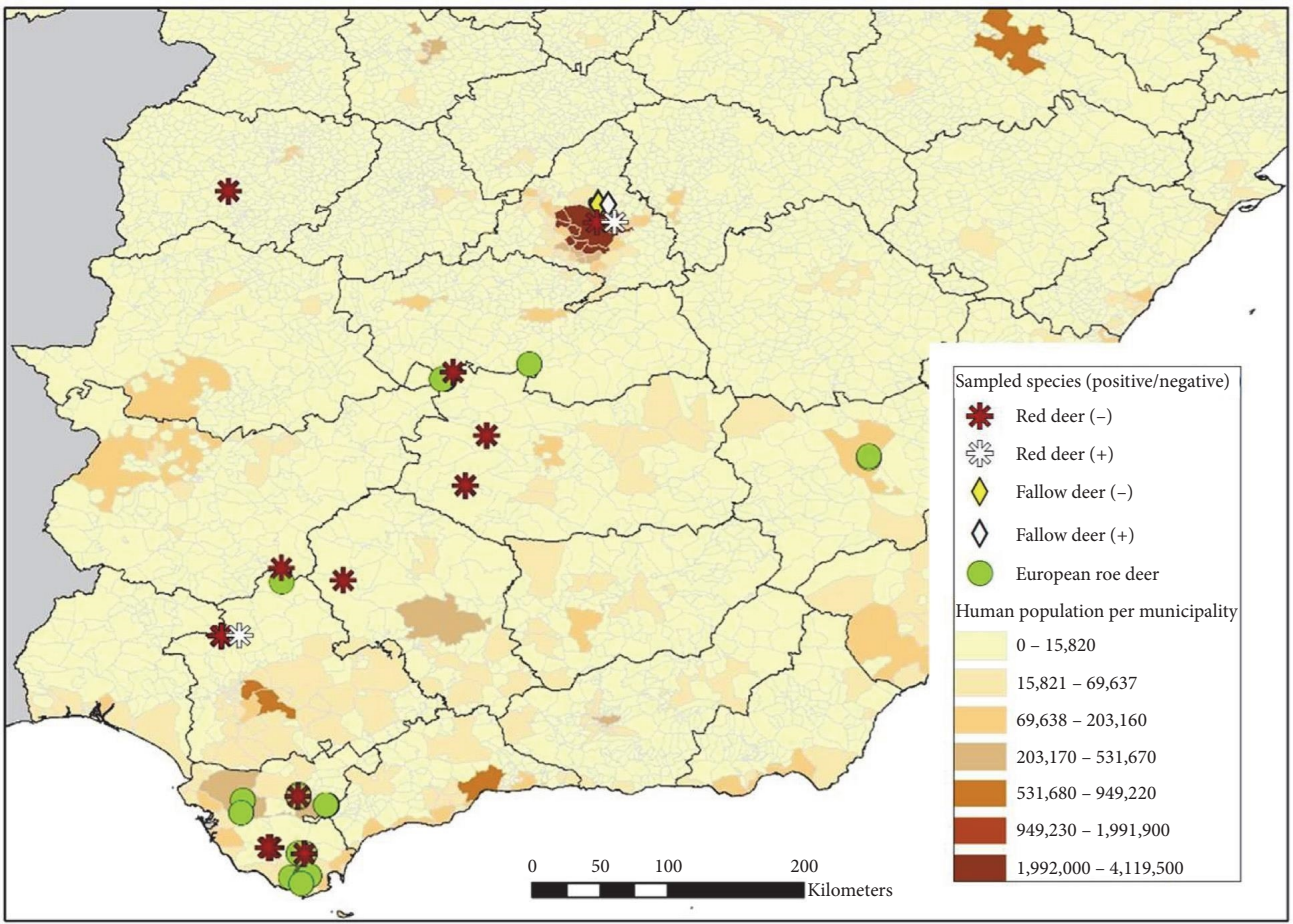
Colocalization of sampling sites of red deer, roe deer, and fallow deer with human populations in Spain. Colors from yellow to brown express increasing density of human population per municipality.

In contrast, all but one of the deer samples (ERD = 71 and RD = 44) also collected during the pandemic period in rural areas with low human density were seronegative (Figure 5).

**Figure 6:**
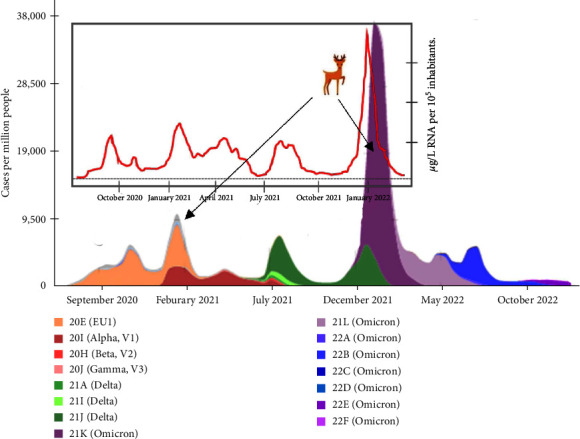
Estimate of cases of SARS-CoV-2 variants in Spain (colored peaks, left axis), concentration of SARS-CoV-2 in wastewater in Madrid (red line, right axis), and sampling date of seropositive deer (black arrow).

The five seropositive samples from Monte de Viñuelas were collected at the end of the peak incidence of COVID-19 in the province of Madrid, with 19,878 clinical cases, 98% of which caused by the Omicron variant on December 23, 2021 (Figure 6). On the same day of collection of positive samples (February 9, 2022), 7,229 clinical cases of COVID-19 were recorded in Madrid [42]. The measurement of the viral load in wastewater is a good indirect indicator that reflects the degree and trend of SARS-CoV-2 circulation in a given place at a certain time [43, 44]. Interestingly, the average virus concentration in the province of Madrid on the sampling date was the highest recorded during the pandemic (Figure 6) [45]. These data confirm that SARS-CoV-2 was circulating intensively in Madrid at the time of sampling. In February 2022, the Omicron variant was predominant and had emerged 2 months earlier in December 2021 (Figure 6) [46]. The peak incidence of the second wave of the Delta variant occurred at mid-December 2021 and decreased to 2% by mid-January 2022, while Omicron reached 98%. Regarding the Alpha variant, the peak incidence of its third wave occurred in mid-July 2021 when the first wave of Delta variant emerged. Taking into account this calendar, the seropositive deer detected in this study could have been exposed to all three SARS-CoV-2 variants because serum samples were collected 7 months, 2 months, and few days after the peak incidence of Alpha, Delta, and Omicron, respectively (Figure 6). It has been demonstrated that specific antibodies against SARS-CoV-2 persist in naturally infected deer for at least 13 months [36]. One of the two seropositive samples from RD (#1367) was collected on February 12, 2021, when the 20E (EU1) variant predominated (72% of the sequenced samples) and Alpha variant accounted for the 28% of the cases in Spain [46]. Sample #1367 only shows a low titer against Delta variant (Figures 3(b) and 3(c)) probably due to cross-reactive antibodies elicited by 20E (EU1) since Delta emerged 4 months later in July 2021 (Figure 6).

It is assumed, as with North American WTD, that the majority (six out of seven) of positive animals found in our study were exposed to SARS-CoV-2 due to relatively high levels of human–deer interactions, which created favorable opportunities for virus transmission from infected people. This situation does not occur in free-ranging cervids in rural areas, which rarely, if ever, come into close contact with people. Therefore, our study supports the accepted hypothesis of WTD infection in North America indicating that SARS-CoV-2 infection of deer may have been transmitted directly from humans. Similarly, our findings show that deer have been exposed to SARS-CoV-2 but do not clarify whether these were single transmissions events from humans or whether the virus had spread among the animals. On the other hand, it is unlikely that the deer had cross-reactive antibodies elicited by other coronaviruses because all samples collected before the pandemic were negative and sera from deer recognize the SARS-CoV-2 variants circulating in nearby human populations fairly well at the time of sampling. It has been claimed that hunting could be a major driver in human-to-deer SARS-CoV-2 transmission in the U.S. [16]. However, in our case, it is rule out because hunting is not allowed in the sampling area, whereas the seronegative samples from rural areas were collected in permitted areas during hunting season.

Indirect human-to-deer transmission, such as involvement of an intermediate animal species or contamination with fomites, would also be possible. Recently, there have been reports of SARS-CoV-2 infection of rodent species that could act as intermediate hosts [47]. Other intermediate hosts as transmitters might be wild minks and feral cats [8, 48]. On the other hand, there is an important social center in Monte de Viñuelas where massive public events are held, which can generate contaminated waste that could be vehicles of infection for animals. The high concentration of SARS-CoV- 2 in wastewater measured in Madrid at the time of sampling of the seropositive animals, in addition to indicating a high circulation of the virus in humans, could be a direct or indirect source of infection.

Our study shows that other cervid species, in addition to WTD, such as FD and RD, can become infected with SARS-CoV-2. However, we did not find any seropositive ERD, taxonomically closer to WTD than FD and RD. It should be noted, however, that there were no ERD in the seropositive sampled area and that all the samples from this species in our study were collected in rural areas with few or no people to interact with. Epidemiological studies carried out in wild ruminants in Spain have shown an effective interspecies transmission of several pathogens between RD, FD, and ERD and have also shown a heterogeneous prevalence of antibodies against different pathogens depending on the animal species and its spatial distribution [49–51]. Interestingly, FD is the most anthropogenically influenced among cervids in Spain and can create very dense populations due to its gregarious behavior [52]. These facts would support the importance of behavioral factors and human intervention activities in the efficiency of pathogen transmission in wildlife. Studies with a larger number of samples or experimental infections would be needed to evaluate the differential susceptibility between the different cervid species. The serological data obtained in this study have provided us with very valuable information for designing a more precise sampling of nasopharyngeal swabs aimed at detection, genetic characterization, and isolation of SARS-CoV-2 circulating in deer in Spain.

The period of confinement in Spain was from March 15, 2020 to June 21, 2020, so from the end onward human contact with animals was possible, although some mobility restrictions remained for a few more weeks. The first postconfinement sampling was carried out in November 2020, 5 months after the end of the lockdown. However, samples collected at that time were negative, possibly because SARS-CoV-2 circulation, at that time the 20E (EU1) variant, was not high enough for transmission and mobility of people was low. It cannot be ruled out, however, that the number of collected sera were not sufficient. In February 2022, the sampling coincided with the peak incidence of Omicron variant and people began to move more freely, thus increasing the likehood of human–animal contact. As mention before, the peak incidence of Alpha and Delta variants in humans in Spain was 7 and 2 months before sampling of the seropositive samples, time enough to mount a significant humoral response in case of SARS-CoV infection of deer with one or both of the variants. Persistence of SARS-CoV-2 neutralizing antibodies has been shown to persist longer than 13 months in naturally infected WTD [35]. However, the peak incidence of Omicron was a few days before the collection of seropositive samples; therefore, in case of infection, there would not be sufficient time for a high production of specific antibodies. The antibody titers of the seropositive samples were measured by cVNT against the Alpha, Delta, and Omicron BA.1 SARS-CoV-2 variants (Figure 3(b)). In five of the six positive samples, the titers showed a similar profile with significant higher values for Delta than for Alpha and Omicron. This would be consistent with Delta being the cause of the primary infections in these animals, as Delta neutralizing antibodies are known to have slightly reduced neutralization against other variants, with a larger drop in the case of the more antigenically drifted Omicron [53, 54]. However, we cannot exclude the possibility that some of the deer might have been infected by more than one variant.

## 4. Conclusions

Our studies show that European RD and FD are susceptible to infection with SARS-CoV-2 and that, as has been shown in WTD in North America, the virus is likely to have been transmitted by infected people in circumstances that favor human–deer interaction. These preliminary results highlight the need for systematic surveillance to elucidate the epidemiological factors involved in the reciprocal human–deer transmission of SARS-CoV-2 in order to implement control measures to prevent these species from becoming natural reservoirs and potential transmitters of SARS-CoV-2 to humans, livestock, and other wildlife species.

## Figures and Tables

**Table 1:** Number (*N*) of serum samples of cervid species (female/male, adult/juvenile) collected in Spain.

	European roe deer (ERD)	Red deer (RD)	Fallow deer (FD)
Prepandemic period (2017–2019)	45 (1/44, 45/0)	19 (17/2, 15/4)	5 (4/1, 3/2)
Pandemic period (2020–2022)	71 (71/0, 71/0)	44 (20/24, 33/11)	31 (20/11, 21/10)

## Data Availability

The data supporting the findings of this study are available on request from the corresponding authors.
